# Advanced Low–Cost Natural Materials for High–Performance Oil–Water Filtration Membranes: Achievements, Challenges, and Future Directions

**DOI:** 10.3390/membranes14120264

**Published:** 2024-12-08

**Authors:** Nthabiseng Ramanamane, Mothibeli Pita, Baonhe Sob

**Affiliations:** 1 Department of Mechanical Engineering, Bioresources, and Biomedical Engineering, College of Science, Engineering and Technology, University of South Africa, Florida 1710, South Africa; pitam@unisa.ac.za; 2 Department of Mechanical Engineering, Mount Vernon Nazarene University, 800 Martinsburg Rd, Mt Vernon, OH 43050, USA; baonhe.sob@mvnu.edu

**Keywords:** ceramic membranes, natural materials, oil–water separation, environmentally friendly, membrane performance, separation efficiency

## Abstract

The development of affordable ceramic membranes is essential for reducing expenses and optimizing the treatment of oily wastewater. There is an urgent demand for membranes that are not only affordable and easy to operate but also stable and capable of managing high fluxes to address the increasing volumes of oily wastewater. The significant production demands associated with many commercially available ceramic membranes, primarily due to the use of specialised raw materials and intricate processing methods, limiting their suitability for many wastewater treatment applications. Consequently, there is a rising interest in creating innovative ceramic membranes using affordable materials and simpler production techniques. This study reviewed the oil–water ceramic membranes utilizing affordable natural ceramic materials aimed at improving membrane performance. It focused on reviewing the environmentally friendly and economically viable membranes derived from natural ceramic resources as an alternative to conventional synthetic membranes. These natural ceramic materials possess crucial properties like hydrophilicity and oleophobicity, which are vital for effective oil–water separation. The ceramic membranes were reviewed for their filtration performance and advantages. It was reported that these natural ceramic material-based membranes demonstrate superior separation efficiency, and strong mechanical stability, making them promising candidates for sustainable water treatment.

## 1. Introduction

The increasing levels of industrial wastewater, particularly oily wastewater, pose a significant environmental challenge [1]. Furthermore, there is evidence of the frequent growth of water demand globally that has driven the research perspective to separation studies [2,3,4]. Effective treatment of this wastewater is essential to preventing the contamination of water bodies and to complying with environmental regulations [5]. Traditional methods for treating oily wastewater, such as chemical treatments and mechanical separation, often fall short due to high costs, complex operations, and inefficiencies. Membrane technologies such as ceramic, polymeric, and composite membranes have emerged as promising alternatives due to their ability to efficiently separate oil from water [6,7]. The widely used materials for the fabrication of oil–water membrane filtration are polymer and ceramics materials. It has been reported that ceramic material offers more separation benefits over polymer material, such as thermal stability; presents excellent mechanical robustness; and can withstand complex oil–water chemical compositions [8].

Despite all the benefits exhibited by ceramic material, it has been reported that the commercial ceramic membrane used for oily wastewater separation are costly compared to polymers due to the high costs of the raw materials and manufacturing processes [9,10]. Therefore, in recent years, the investigations of abundant ceramic membrane or membrane supports developed from natural material such as kaoline, bauxide, quartz sands, and clays became more important for oily wastewater filtration manufacturing [11]. Several studies reported that employing the natural ceramic material into the manufacturing of the low-cost ceramic membrane will not only reduce the raw material costs but will equally reduce the manufacturing-energy-related costs by reducing the sintering temperature [11].

Low-cost ceramic membrane materials have been reported as the promising materials for oily wastewater separation due to their greatest influence on membrane separation efficiency and increased permeability in addition to the existing benefits reported [12]. Unlike natural ceramics, which are derived from naturally occurring minerals such as kaolinite and quartz, synthetic ceramics are engineered materials specifically designed to exhibit superior and customizable properties. These materials are manufactured through controlled chemical processes to enhance characteristics like thermal resistance, mechanical strength, and chemical stability.

Therefore, this paper reviewed the oil–water filtration membranes using natural ceramic materials to enhance performance of the membrane. By focusing on environmentally friendly and economically viable solutions, the review addressed the limitations of conventional synthetic membranes to offer a sustainable alternative for oily wastewater treatment. The review further focused on the challenges faced in the development of these ceramic membranes and highlights the successes achieved in creating functional, durable, and efficient filtration systems.

## 2. Membrane Technology Used for Oil/Water Filtration

**Figure 1:**
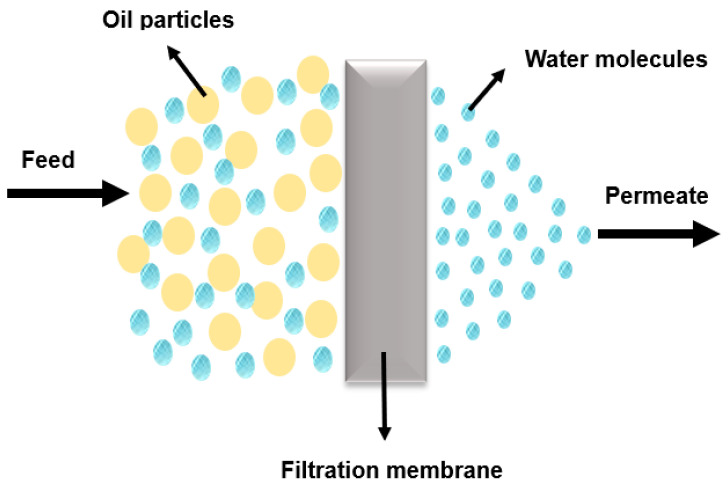
Oil and water filtration membrane.

Membranes used in oily wastewater treatment are generally classified into two main categories based on their material composition: organic (polymeric) and inorganic (ceramic, metal, glass, etc.) [13]. In general, membrane technology for oil/water separation relies on surface selectivity tailored to the desired end product. Figure 1 illustrates a general demonstration of membrane functionality in oil/water separation.

The wide variety of materials allows customization based on specific process conditions, such as high temperatures, where ceramic or metallic membranes often outperform other options [14]. One of the key factors classifying membranes used in this process is their pore size, which determines the separation efficiency for oil and water. The pore size influences the ability of the membrane to reject oil droplets, suspended solids, or other contaminants while allowing clean water to pass through. Table 1 provides a basic overview of pore size classifications commonly employed in oil/water separation applications.

The diversity in membrane materials broadens the scope for membrane technology, enabling it to compete with alternative processes like chemical treatments, skimming, adsorption, flotation, etc. However, significant challenges remain, particularly in terms of membrane fouling [15,16]. Fouling, a major issue across all membrane types, leads to performance degradation over time, necessitating frequent cleaning operations and resulting in increased operational and capital costs [17,18]. The hydrophobic nature of polymeric membranes is noted as a key disadvantage, making them more prone to oil adherence and fouling compared to ceramic membranes [19,20]. In contrast, ceramic membranes exhibit superior chemical and thermal stability, resistance to fouling, and longer lifespans, making them more suitable for harsh environments [21,22].

Similar to other studies in the field, this review underscores that both organic and inorganic membranes require careful material selection to optimise performance for specific applications. The selection of membrane material is critical for achieving desired properties like permeability, rejection rate, and stability under varying operational conditions [23]. The problem of membrane separation for oil and water has garnered significant attention in literature due to its critical applications in industries such as wastewater treatment, petroleum refining, and food processing. While membrane technology offers an efficient and environmentally friendly solution, the persistent challenge of **membrane fouling** remains a significant barrier to its widespread adoption and optimal performance. Numerous studies have highlighted the excellent performance of ceramic membranes in oil/water separation. Given the critical role that membrane material selection plays in achieving effective separation, the membrane materials are summarised in Table 2, providing guidance for optimal selection across a range of applications.

Youssef et al. [39] fabricated a ceramic membrane with optimised porosity and pore size, achieving a high permeability of 959.64 $L\cdot m^{-2}\cdot h^{-1}$/bar. The study highlighted the role of precise control over membrane microstructure in achieving high flux. Similarly, Wirginia et al. [40] reported a 30% improvement in permeate flux by varying the transmembrane pressure of a ceramic membrane system. For example, Amos et al. [41] achieved a water flux of 303.63 $L\cdot m^{-2}\cdot h^{-1}$/bar using an ultrafiltration ceramic membrane, while Zhaoyubo et al. [42] reported a consistent flux of 305 $L\cdot m^{-2}\cdot h^{-1}$ and a 95% oil rejection rate.

Studies by Yijiang et al. [43] and Mehrdad et al. [44] demonstrate that ceramic membranes can achieve oil recovery efficiencies as high as 98%. This aligns with the broader consensus in the literature that ceramic membranes offer excellent performance in oil rejection. Nafiu et al. [23] highlight the significance of selecting appropriate materials for fabricating oil/water filtration membranes, suggesting that material properties are the primary determinants of membrane performance.

This view is consistent with other research, which emphasises the need for materials that balance hydrophilicity, oleophobicity, and mechanical strength to ensure high separation efficiency. A critical comparison of the emerging natural ceramic membranes alternatives would provide a more comprehensive understanding of sustainable membrane used for oil/water separation.

## 3. Benefits and Drawbacks of the Commercial Ceramic Membranes Used for Oily Wastewater Separation

Ceramic membranes, derived from inorganic materials, are widely recognised as one of the most promising technologies for the treatment of industrial oily wastewater [45,46]. Their advantages include excellent mechanical, chemical, and thermal stability, which makes them well-suited for harsh conditions where organic membranes might fail. However, despite these strengths, the performance of ceramic membranes in oil/water separation is significantly hindered by issues like membrane fouling and pore blockage, which result in a decline in filtration efficiency over time [47]. Although the literature acknowledges the issue of fouling, many studies focus primarily on the initial performance of ceramic membranes without offering detailed long-term evaluations of how fouling impacts their effectiveness. There is a lack of comprehensive studies on the sustainability of fouling mitigation strategies and their economic feasibility over extended periods of use. Research by Yusuf et al. [48] demonstrated that membrane fouling could dramatically decrease oil flux performance, with a reduction from 37.02 $L\cdot m^{-2}\cdot h^{-1}$ to 2.50 $L\cdot m^{-2}\cdot h^{-1}$ observed during testing.

Dionisio et al. [49] also highlighted the challenges of fouling when varying oil concentrations in experimental setups, reporting a significant drop in permeate flux due to pore blockage. While ceramic membranes offer superior chemical and thermal stability compared to polymeric membranes, their high susceptibility to pore blockage presents a critical drawback in practical applications. For instance, standard microfiltration membranes with a pore size of 0.55 µm, as studied by various researchers [50,51], show reduced flux during the filtration of oil/water emulsions, leading to the formation of a cake layer that further impedes fluid flow.

Several studies agree on the mechanisms of flux decline in ceramic membranes, often pointing to similar fouling stages such as standard blocking, complete blocking, intermediate blocking, and cake layer formation [52]. This similarity indicates a well-documented pattern of performance decline, yet there is limited innovation in the field regarding new materials or surface treatments to significantly reduce these effects. The focus on ceramic membranes’ stability and fouling resistance tends to overlook other critical factors, such as the accessibility of raw materials and the energy-intensive nature of their production. This makes ceramic membranes less economically viable for large-scale or low-budget applications. The high production implications are often due to the need for expensive raw materials and complex sintering processes. As a result, their use is often restricted to applications in which long-term durability outweighs initial investment [53,54].

Given the limitations of traditional ceramic membranes, recent research has shifted towards exploring more affordable alternatives. Studies have focused on using natural materials like kaolin, clays, quartz, and industrial waste products such as rice husk ash, and fly ash as raw materials for ceramic membrane production [55,56]. These alternatives aim to develop affordable membranes while maintaining acceptable performance levels. For example, naturally abundant clays have shown promise due to their intrinsic hydrophilic properties, which can aid in reducing oil fouling. Most studies focus on laboratory-scale experiments without exploring how these materials behave under fluctuating operational pressures or varying wastewater compositions encountered in industrial settings. The push for more economical and sustainable materials for oil/water separation highlights the dual challenge of developing affordable membranes while maintaining or improving performance.

While alternatives like kaolin and quartz are abundant, their long-term durability and ability to withstand harsh chemical environments are not yet fully understood. Ceramic membranes offer a range of advantages, including high thermal and chemical stability, making them well-suited for challenging wastewater environments. However, their susceptibility to fouling and regular maintenance remain significant barriers to widespread adoption. As a result, there is an increasing demand for membranes that leverage natural, abundant materials.

## 4. Natural Materials for Developing Oil and Water Filtration

In recent years, the focus on affordable ceramic materials has gained significant traction among researchers aiming to develop more economical and efficient membranes for treating oily wastewater. This research is particularly crucial given that around 2.5 billion people in developing regions lack access to clean water [57]. The situation is exacerbated by the rise in industries such as petrochemical, oil and gas, food, and beverages, which contribute to an increasing volume of oily wastewater and heightened water scarcity [58]. Abundant and affordable ceramic membranes have emerged as a promising solution, capable of providing large-scale filtration of oily wastewater, thereby offering clean water to underprivileged communities [59]. Consequently, there is a growing interest in using naturally abundant and affordable materials for the development of ceramic membranes.

**Figure 2:**
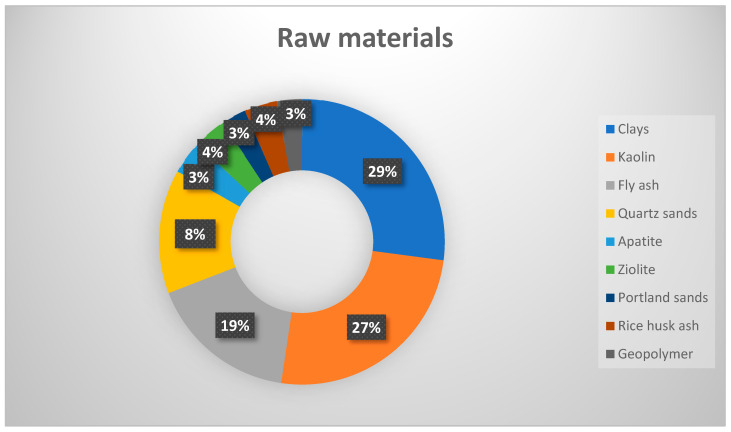
Samples of raw materials used for fabrication of oil and water filtration membrane [67].

Various natural and abundant materials, such as clays, zeolites, quartz, apatite, and industrial by-products like fly ash and rice husk ash, as indicated in Figure 2, have been studied as base materials for oil and water filtration membranes. These materials are not merely crushed and ground but are further processed to meet the structural and performance requirements for membrane applications. The advantage of these materials lies in their availability and affordability, but their mechanical and chemical stability under varying operational conditions requires further investigation.

A wide range of other clay types are also of interest for affordable oil/water separation membranes, in addition to kaolin. Sepiolite, ball clay, bentonite, and attapulgite are some of these clays. Another technique that shows promise is membrane preparation using attapulgite clay for the oily wastewater filtration process [60]. One of the most significant naturally occurring fibrous clays is attapulgite, which has a number of desirable qualities, including a large specific surface area, robust mechanical strength, great adsorptive capacity, and high chemical and thermal stability [61]. Additionally, attapulgite can be used to create oily wastewater separating membranes without the necessity for extreme temperature sintering [62]. Attapulgite-based oily-wastewater-separating membranes, which compete with conventional membranes in UF applications due to their fibrous nature, offer competitive mechanical and filtering capabilities, with pore diameters of about 12 nm and permeability above 60% [63].

Since clays are mineral combinations, it is difficult to conduct a comparison examination because the composition of different clays depends on their geographic origin [64]. Clay-based supporting layers that have been developed for use in oil and water separation membranes typically feature pores that range in size from 0.3 µm to 16 µm, a permeability of up to 49%, and flexural strengths of 10 MPa to 69 MPa [65]. It has been proven that clays can be used as source materials to create MF or UF layers that are active for applications such as suspended particles, oil droplets, dye, or heavy metal extraction [66]. According to the results thus far, clay can be used to manufacture membranes in the context of environmentally friendly oil and water filtration technology.

### 4.1. Analysis of Kaolin Material Used to Develop Oil/Water Membranes

Kaolin, a type of clay that contains kaolinite as its principal mineral, is particularly favored for membrane production due to its beneficial mechanical and thermal properties. Different clay types, including kaolin, are defined by their unique physical, chemical, and mechanical characteristics, which depend on their formation conditions [68]. Kaolin is abundant globally and can be processed into membranes with minimal effort, making it a key candidate for affordable membrane technology [69]. Kaolin stands out among clays due to its ability to form membranes with desirable pore structures and mechanical strength after heat processing of about 1200 °C. Unlike more traditional ceramic materials, kaolin can achieve effective performance at relatively lower sintering temperatures, which makes production affordable. However, when compared to synthetic ceramics, kaolin-based membranes may have limitations in terms of consistency and quality control, especially when sourced from different geological locations. While the lower processing temperature of kaolin is advantageous, there data on how kaolin membranes perform in treating wastewater with varying oil concentrations or under fluctuating pressure conditions typical in industrial settings are limited.

Omar et al. [70] conducted extensive research on kaolin-based membranes, focusing on their fabrication and affordability. They highlighted the decomposition of kaolin into phases like spinel and mullite at lower temperatures than those required for other ceramics, which is a key factor in the affordability of these membranes. Kaolin membranes have been developed both as single-component systems and composites [71]. Research has shown that sintering kaolin at temperatures above 1200 °C without additives (Alumina, Silica, Titania, and Zirconia) results in the formation of mullite and cristobalite, which can enhance membrane strength [72]. However, achieving small pore sizes and high mechanical stability often necessitates the addition of reactive materials to reduce sintering temperatures and control pore structure [73].

While the addition of additives (Alumina, Silica, Titania, and Zirconia) can reduce the sintering temperature, it introduces complexities in the production process that may offset some of the advantages offered by kaolin. Additionally, the need for the precise control of additive concentrations and thermal conditions presents a challenge for maintaining uniform membrane properties, particularly in larger batches. This is a significant gap in the current literature, as there is a lack of guidelines or standardised methods for optimizing these parameters during kaolin membrane production. Studies have shown that kaolin-based membranes can achieve comparable performance to commercial synthetic membranes, with porosity levels between 30% to 50% and pore sizes ranging from 0.1 to 1.2 µm [74,75]. This makes them viable for applications such as microfiltration and ultrafiltration.

However, their practical deployment is limited by a lack of comprehensive performance data in real-world scenarios. For example, there is insufficient information on their resistance to chemical corrosion or their durability when exposed to prolonged operational cycles. Like other natural materials used in membrane production, kaolin benefits from being widely available and relatively easy to process. Its use aligns with broader efforts to create more sustainable filtration technologies. However, similar to other natural materials, its performance can be highly variable depending on the specific properties of the raw material, such as mineral composition and particle size. This highlights the need for further research into standardizing raw material selection and processing methods to ensure consistent membrane quality. The development of affordable ceramic membranes using natural materials like kaolin presents a promising path toward addressing global water scarcity, especially in regions where access to clean water is limited. However, the transition from laboratory-scale research to industrial applications faces several challenges. These include the need for standardised processing techniques, a deeper understanding of long-term membrane stability, and more robust performance testing under real-world conditions. Addressing these gaps will be crucial for making natural-material-based membranes a viable alternative to conventional synthetic membranes in the field of oily wastewater treatment.

### 4.2. Zeolite Raw Materials Impact on Oil/Water Filtration Membrane

Zeolites are found in many different types of rocks, but volcaniclastic sediments contain most of them. Alternated vitric tuffs have the biggest and cleanest zeolite deposits. Zeolites are used in a broad variety of fields, including agriculture, catalysis, construction, oily wastewater treatment, and medicine [76]. There are numerous varieties of zeolites made from synthetic materials that can be produced using hydrothermal processes or other appropriate techniques. However, this thesis focused primarily on naturally occurring zeolite materials, which are more directly applicable to the production of low-cost oily wastewater filtration systems and can be supplied cheaply in large quantities. Natural zeolites must be crushed, shaped, and sintered in order to provide sturdy bulk materials with the necessary aluminosilicate phases for the manufacturing of oil and water separation membranes [77,78]. Natural zeolite was utilised in a previous research study by Jiang et al. [79] to create permeable support layers for oily wastewater filtration membranes. Using various ground zeolite particle sizes, multi-layer ceramic microfiltration membranes were subsequently created. The hole diameters of the obtained membranes ranged from 0.3 to 1.1 µm.

Pores that are as large as 6 µm can be produced by utilizing starch as a pore-forming agent. Unless pore-forming chemicals are used, zeolites sinter at relatively low temperatures, in the range of 800–900 °C, after being fired at 1000 °C. Additionally, the naturally occurring pores in the zeolite structure may be filled with liquid phases, removing permeability [80]. Zeolite-based oil/water separation membranes’ mechanical strength has not yet been thoroughly investigated [81]. According to Yanan et al. [82], a three-point bending test revealed a strength of up to 50 MPa for hollow fibre zeolite oil/water separation membranes.

Even so, this hollow fibre oil/water separation membrane has a good flexural strength when compared to others; for instance, kaolin-based hollow fibre oil/water separation membranes have flexural strengths of 15–63 MPa at sintering temperatures of 1200–1500 °C, whereas the zeolite–based oil/water separation membrane’s sintering temperature is 1050 °C. Hollow fibre ceramic membranes with separation–adsorption dual properties, i.e., the ability to adsorb chromium and ammonia ions while providing filtration, were presented by Yanan et al. [83] in a study on the manufacture of ceramic membranes employing zeolite. Zeolites are well recognised for their high ammonia ion absorption, which makes them useful for applications in the treatment of water contaminated with oil. Although zeolite membranes demonstrate promising filtration capabilities, gaps exist in fully understanding their mechanical resilience and durability under varying operational conditions.

Most studies do not systematically assess the impact of different particle sizes, sintering temperatures, and pore-forming agents on membrane performance. Furthermore, natural zeolites’ inherent variability makes reproducibility a challenge, requiring further research into optimizing processing methods for consistent results. Compared to traditional ceramic membranes, zeolite-based membranes offer lower sintering temperatures (800–1050 °C), reducing energy consumption. However, they lack the high-temperature stability of conventional alumina membranes, which limits their application in aggressive conditions. Like other mineral-based membranes, zeolites can adsorb ions, enhancing their utility in applications beyond simple filtration, such as heavy metal removal.

### 4.3. The Use of Apatite Materials of Fabricate the Oil/Water Filtration Membranes

Apatite materials are organic substances with numerous uses in the geological, chemically based, and environmental domains. Numerous studies have been done on the use of apatite material in membrane technology to treat various oily wastewater solutions. Apatite materials are effective at removing oil emulsions through adsorption in the membrane in addition to efficiently adsorbing oily wastewater impurities. Consequently, the use of apatite materials in adsorption membrane systems for treating oily wastewater has been the focus of most investigations [84]. Nevertheless, one of the earliest works on the use of apatite material as an oily wastewater treatment membrane was a study by Sun et al. in which apatite was utilised as a low-cost raw material to create oily wastewater membranes. Similar results were revealed regarding the dose of zeolites: that apatite membranes are useful for attaining bidirectional adsorption–filtration capacities that make it easier to remove several types of oily wastewater contaminants in one process [85].

According to research currently available, naturally occurring minerals are more affordable to use in the production of apatite-based oily wastewater membranes. Findings revealed that different particle sizes of membranes made from natural apatite materials display similar characteristics and performance to membranes made from synthetic materials. Furthermore, the fabrication of flat support layers employing apatite was reported by Conidi et al. [86], and the resulting materials showed a flexural strength of up to 30 MPa after being sintered at a temperature of 1210 °C. Apatite’s mechanical strength is equivalent to that of other raw material membranes used for oil/water separation made of inexpensive materials.

Despite the promising performance of apatite in adsorption and filtration, the field lacks detailed studies on its mechanical stability over extended periods and under varying operational conditions. Furthermore, the comparison between apatite and other potential materials, such as pozzolan or bauxite, remains underexplored, limiting the broader applicability of apatite in diverse industrial scenarios. Similar to zeolite, apatite excels in its dual adsorption–filtration abilities, particularly for ion exchange applications. However, it generally requires higher sintering temperatures (around 1210 °C), which could increase production costs. Comparative studies between apatite and zeolite could provide deeper insights into their respective advantages for specific wastewater treatments.

### 4.4. Analysis of Pozzalan Materials Employed to Manufacture Oil/Water Filtration Membranes

When lime and water are present, materials with components that mix with lime at room temperature to generate permanently insoluble and stable compounds that behave like cement are referred to as pozzolans. This explains why the cement industry mostly uses organic pozzolan materials. In order to create MF membranes with a permeability of about 30% and pore diameters of 2–3 µm that were effectively used for treating oily wastewater from textile industries [87]. Yang et al. fused pozzolan material at 950 °C rather than making a cement. Subsequently, the impact of starch serving as a pore-forming reagent was investigated by Lesak et al. and it was discovered that it is feasible to improve permeability in these inexpensive oil/water-separating membranes by up to 50% [88]. For the processing of oily wastewater treatment operations, for a support layer of graphene oxide compound membranes, as well as a support layer for synthetic zeolite membranes, tubular pozzolan multi-layer oil/water separating ceramic membranes were created in previous works by Sun et al. [89].

The positive preliminary findings encourage additional research into membranes based on pozzolan used for the oil and water separation process. However, 950 °C has been employed as the sintering temperature in all the research that has been published on this subject [90]. To find out if durable and useful phases form at higher temperatures or how permeability and distribution of pore sizes vary with thermal treatment and density, phase change behaviour with temperature should be researched. Therefore, it is essential to further understanding how other low-cost raw materials, such as bauxite, perform during oil and water filtration testing. Research on pozzolan-based membranes has largely focused on low-temperature sintering. However, the effects of higher temperatures on phase stability, pore structure, and mechanical strength are not well understood. Future studies should investigate whether higher sintering temperatures could enhance membrane durability without compromising permeability. Unlike zeolite and apatite, pozzolan materials benefit from lower production costs and accessibility, making them attractive for large-scale applications. However, their performance is generally lower in terms of adsorption properties, suggesting that they may be best suited as support layers rather than active filtration components.

### 4.5. Evaluation of Bauxite Materials Used to Develop Oil/Water Filtration Membrane

The global resource of bauxite, a type of sedimentary rock mostly composed of aluminium mineral substances, is believed to be between 55 and 75 billion tons. Alumina, along with a few other iron and silicon oxides, makes up the majority of the bauxite residue at high temperatures. Alumina is frequently used to prepare conventional membranes, and oily wastewater membrane operations may be interested in bauxite, an alumina precursor that has specific pollutants. Wang et al. [91] investigated phase reversal through immersion and subsequent high-temperature sintering processes for the manufacturing of hollow fibre membranes used for the oil and water separation process. When compared to pure alumina hollow fibre membranes, the results from Wang’s findings indicated a flexural strength between 24 and 183 MPa at sintering temperatures between 1200 °C and 1350 °C. In contrast, despite a higher sintering temperature ranging from 1250 °C to 1450 °C, Oluwaseun et al. [92] reported that the membrane’s flexural strength was between 5 and 70 MPa.

Although the resulting membranes’ permeability and pore size have not been thoroughly investigated, images from scanning electron microscopy indicate that their morphological qualities are competitive with those of membranes made of pure alumina [93]. In general, the idea of creating membranes solely from bauxite is advantageous and effective because it does away with superfluous steps like collecting alumina first and then creating a membrane [94]. However, exploring the impact of sintering temperature, fabrication methods, and beginning bauxite particle size on the mineralogical composition, shape, and oil/water filtration capabilities of membranes containing just bauxite requires extensive research [95]. Additionally, low-cost materials like quartz would be beneficial for understanding the properties of commonly used raw materials in fabricating oil and water filtration membranes.

Despite the promising strength characteristics, the permeability and pore size distribution of bauxite membranes have not been comprehensively studied. Further research is necessary to determine the optimal particle size and sintering conditions for achieving the desired filtration performance. Bauxite membranes offer similar mechanical strength to alumina-based membranes but require fewer processing steps, reducing overall production complexity. However, they necessitate higher temperatures compared to zeolite or pozzolan, which could increase energy consumption.

### 4.6. Impact of Quartz Membrane Materials Used to Develop Oil/Water Filtration Systems

Natural quartz particles are sedimentary rocks made up of quartz-like crystalline silicon dioxide. Quartz material has a high level of resistance to weathering, both mechanically and chemically [96,97,98]. As a result, quartz is one of the most prevalent and widely dispersed minerals on Earth’s surface. On rare occasions, quartz-dominated sands have been created by geological processes. The quartz material is mostly employed by the glassmaking sectors. Quartz material has been utilised in oil/water filtration purposes for quite a while [99,100]. Slow quartz sand filters (SSF), an older industrial oil/water treatment technology, were first created in 1829 [101]. As the title suggests (slow quartz sands filtration), this type of oil and water purification is labour-intensive and has numerous disadvantages, which is why slow-moving sand filters are now primarily used in developing nations [102]. A binder phase is necessary for the production of quartz-particle-based membranes because it helps the quartz particles adhere to one another, ensuring acceptable performance and robustness during oil and water separation [103,104]. When appropriate binders are selected, it is possible to create performing quartz-particle-based membranes used for oil/water separation at temperatures as low as 600 °C.

However, in order to obtain reinforcement layers with the requisite mechanical strength, additive-assisted sintering of quartz generally requires temperatures of at least 800 °C [85]. It is necessary to sinter quartz material at temperatures greater than 1040 °C in the absence of sintering additives. It is important to note that materials made from composites predominantly constituted of quartz particles have sufficient strength at comparatively low temperatures, often between 12 and 20 MPa, with a sintering temperature of roughly 1200–1300 °C [105,106]. According to a review of the literature, it is possible to create microfiltration and ultrafiltration supporting layers for oily wastewater filtration membranes using naturally occurring, inexpensive quartz particles [107,108].

To fully comprehend the impact of sintering temperature, particle size, additives, and pore structure on pore structure and mechanical characteristics, additional thorough research is required [109]. To fully understand the mechanical characteristics of affordable materials currently used for oil and water separation, and to aid in material selection, it is important to compare the low-cost materials used in fabricating oil and water filtration membranes. Current studies focus primarily on the basic sintering processes for quartz-based membranes. Further investigation into the role of particle size, sintering temperature, and additive effects on mechanical properties and pore structure is essential for advancing their use in oil–water filtration. Quartz membranes, like zeolite and apatite, can offer mechanical robustness. However, they are often less effective in adsorption processes, making them more suitable as support layers rather than active filtration layers. Compared to other materials, quartz requires careful selection of binders to ensure adequate performance, adding complexity to the fabrication process.

This review highlights the potential of various natural materials—zeolite, apatite, pozzolan, bauxite, and quartz—in the development of oil–water filtration membranes. Each material presents unique strengths, such as zeolite’s adsorption capacity, apatite’s dual filtration–adsorption properties, and the mechanical robustness of bauxite and quartz. However, gaps remain in standardizing processing methods, understanding the impact of sintering temperatures, and exploring the synergies between these materials. Future research should focus on comparative studies and optimization techniques to leverage the full potential of these natural resources in creating cost-effective and efficient filtration systems. By addressing these gaps, it will be possible to identify the best materials for specific applications, ensuring sustainability and performance in oil–water separation technologies.

### 4.7. Sintering Temperatures of Raw Material

The sintering temperatures, mechanical and chemical resistance, strength, and surface permeability of the above-mentioned raw materials were reviewed for oil/water separation membrane. In the literature, zeolite raw material was reported to have a sintering temperature between 800–900 °C and three-point bending showing strength of 50 MPa, which—when compared to the sintering temperatures of other raw materials, such as kaolin, apatite, pozzolan, bentonite, quartz, and other clays—is relatively low [110,111,112]. In contrast, kaolin raw material, the most common clay variety of which kaolinite is the main mineral form, is particularly suitable for oil/water membrane manufacture due to the pore designs and mechanical qualities that may be achieved after heat processing, although the sintering temperature (1200 °C) is slightly higher than that of zeolite [113,114].

Although kaolin raw material is indicated as a suitable material for oil/water separation membranes, there has been no evident research on the investigation of flexural strength, which compromises the employment of kaolin raw material in the oil/water producing industries [115]. The apatite raw material was reported to have equivalent strength as compared to that of other raw materials, although it is sintered at a higher temperature (1210 °C) than zeolite and kaolin [116,117]. However, to date, there has not been a bending or strength flexural test conducted on apatite raw material [118]. In comparison to bauxite and quartz materials, it was reported that bauxite’s mechanical properties are similar to those of the other raw materials used for the development of oil/water separation [119,120]. The bauxite material exhibited an excessive temperature of about 1250–1450 °C and a flexure strength of between 5 and 70 MPa as compared to the other material used to develop oil/water filtration membranes [121,122,123]. In contrast, quartz material’s sintering temperature is roughly 1200–1300 °C, with a particle strength of between 12 and 20 MPa [124].

Due to its improved mechanical and thermal durability, quartz material has been touted as a suitable raw material for the development of oil/water separation membranes [125,126,127]. Quartz is a typical and widely distributed mineral that is affordable and environmentally friendly; has great purity, chemical uniformity, and thermal stability; and has a high level of resistance to weathering both mechanically and chemically [128,129,130]. Quartz materials are reliable and promising materials that provide optimum wettability and performance during the oil/water separation process; the table of materials is given in Table 3 [131,132].

## 5. Applications of Clays in Oil/Water Membrane Filtration

In addition to kaolin, a variety of other clays have attracted interest for their potential use in low-cost oil–water separation membranes. Clays such as sepiolite, ball clay, bentonite, and attapulgite have been explored as viable alternatives due to their unique properties. Among these, attapulgite has emerged as a particularly promising candidate for oily wastewater filtration. Its natural fibrous structure offers several advantages, including a high specific surface area, strong mechanical strength, excellent adsorptive capacity, and substantial chemical and thermal stability [140]. Unlike other ceramics, attapulgite does not require extreme temperature sintering, making it a more energy-efficient option for membrane fabrication [141]. Despite the potential of attapulgite, current research often lacks a detailed comparison of its performance against other clays like bentonite and sepiolite under identical conditions. This limits the understanding of its relative advantages or drawbacks, particularly when dealing with complex industrial wastewater compositions. Additionally, there is a need for more long-term studies that assess the durability and consistency of attapulgite membranes under varying operational pressures and contamination levels.

Attapulgite-based membranes are competitive with conventional ultrafiltration membranes, especially due to their fibrous structure, which contributes to enhanced mechanical and filtration properties. These membranes have shown the ability to achieve pore diameters as small as 12 nm and oil rejection efficiency rates exceeding 60% [142]. To enable oil and water filtration, researchers have explored various affordable alternatives to traditional components, such as alumina or zirconia [143]. These economical materials include industrial byproducts (such as ash) or natural minerals (like clays, zeolite, quartz, and apatite) [136,144]. Quartz sands stand out among other available raw materials due to their remarkable resistance to weathering, both chemically and mechanically [145,146]. Consequently, quartz sands are among the most abundant and widely distributed minerals on the Earth’s surface. Research indicates that it is feasible to fabricate microfiltration membranes with pore sizes as small as 10 µm using quartz sand with diverse particle sizes [147]. Additionally, membranes for ultrafiltration can be crafted using minute particles with pores as tiny as 10 nm [148].

Panyang et al. [149] explored the use of affordable fly-ash-based material for developing an oil and water filtration membrane. The results revealed a high separation efficiency of ≥98.2%. Similarly, Yan et al. [26] manufactured an oil and water filtration membrane using fly ash as a raw material, sintering it at a temperature of 1050 °C, with a porosity of 38% and pore sizes around 0.4 µm. The findings revealed a membrane permeability of $1.06\times 10^{-13}\;m^{3}{\cdot h}^{-1}{\cdot bar}^{-1}$. Additionally, Deqi et al. [150] noted that the raw material exhibited favourable recyclability and corrosion resistance. Moreover, it offered a novel environmental conservation approach for treating oil-in-water emulsions, aligning with sustainable development goals.

Fayçal et al. [151] produced a membrane suitable for oily wastewater treatment using raw materials consisting of natural diatomite and alumina, featuring pore sizes of approximately 7 µm and a porosity of 46%. The results demonstrated an enhanced water permeability of around 15 $m^{3}{\cdot h}^{-1}{\cdot bar}^{-1}$. Abdul et al. [152] developed a new low-cost membrane for treating oily wastewater using raw materials, quartz sands with uniform pore sizes of approximately 3.5 µm. The test rig varied pressure to achieve optimal conditions for successfully recovering clean and potable water from oil-contaminated produced water by the petroleum industry. The results indicated that the highest pure water permeate flux reached 250 $L/m^{2}\cdot h$ at 4 bars, with a corresponding separation efficiency of >98%. Additionally, stability testing showed a consistent separation efficiency of >96% and a permeate flux of 58 $L/m^{2}\cdot h$ at 2 bars.

**Figure 3:**
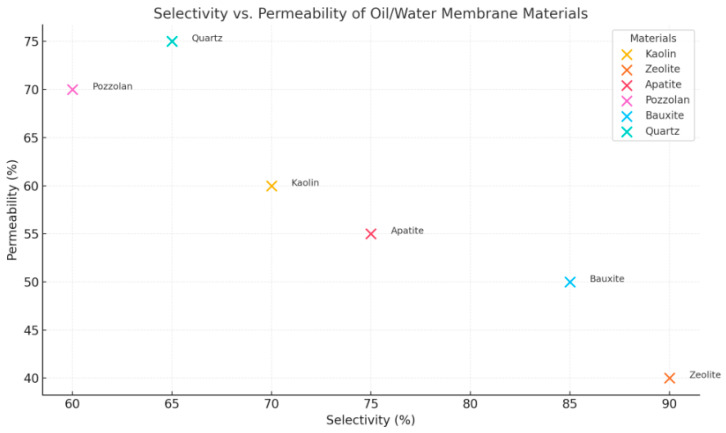
Illustrates the relationship between selectivity and permeability across various oil/water membrane materials [154].

The study concluded that quartz sands have the potential to be a viable candidate for recovering pure water from oily wastewater produced by industries due to their low fabrication and operational costs [152,153]. The reviewed materials each present unique strengths and weaknesses. Quartz membranes offer affordable solutions with high separation efficiencies, while diatomite and alumina combinations provide enhanced permeability. Figure 3 shows the relationship between selectivity and permeability for various oil/water membrane materials (kaolin, zeolite, apatite, pozzolan, bauxite, and quartz). Each point represents a material, with its position determined by hypothetical values for selectivity and permeability, where selectivity indicates the material’s ability to separate oil and water effectively. Permeability reflects the ease with which water flows through the membrane.

Research indicates that clay-based (quartz) support layers used in oil–water separation membranes often exhibit pore sizes ranging from 0.3 µm to 16 µm, permeability of up to 49%, and flexural strengths between 10 MPa and 69 MPa [155]. These characteristics position clays as effective materials for creating microfiltration or ultrafiltration layers, capable of filtering out suspended particles, oil droplets, dyes, and even heavy metals [105].

Clays offer an environmentally friendly alternative for oil–water filtration technologies, as they are natural and often require less energy-intensive processing compared to traditional ceramic materials. This aligns with global efforts toward sustainable industrial processes. Clays, including pozzolan, quartz, kaolin, apatite bauxite and zeolite, hold significant promises for developing affordable and environmentally sustainable membranes for oil–water separation. Their natural availability, low energy requirements for processing, and versatile mechanical properties make them suitable for applications ranging from microfiltration to ultrafiltration. Future research should also focus on developing hybrid materials that combine the strengths of these raw materials to create membranes with enhanced mechanical strength, durability, and filtration efficiency.

## 6. Future Directions

The use of ceramic membranes derived from natural materials represents a significant advancement in oil–water filtration technology. These membranes address key limitations of conventional ceramic membranes, such as high production costs and complex manufacturing processes, by utilizing affordable and readily available raw materials like clays, pozzolan, quartz, kaolin, apatite, bauxite, and zeolite. These natural materials exhibit essential properties like hydrophilicity and oleophobicity, enabling effective separation of oil from water. Moreover, their mechanical stability, environmental friendliness, and cost-effectiveness position them as strong candidates for sustainable wastewater treatment solutions. This review highlights that ceramic membranes based on natural materials not only achieve high separation efficiencies but also align with global goals for environmentally sustainable industrial practices. Despite these achievements, several challenges remain in the development and optimization of natural-material-based ceramic membranes.

Future research should prioritise the following areas:

- Hybrid Membrane Development: The creation of composite or hybrid membranes combining the strengths of various natural materials can enhance mechanical strength, chemical resistance, and separation efficiency.
- Scale Up Production: Exploring scalable, energy-efficient, and cost-effective fabrication methods is critical to enabling widespread adoption of these membranes in industrial applications.
- Performance Optimization: Further investigation into modifying surface properties, such as increasing hydrophilicity and oleophobicity, can improve selectivity and fouling resistance under operational conditions.
- Long-Term Durability Studies: Comprehensive testing of these membranes under real-world conditions, including exposure to harsh chemicals and varying temperatures, is essential to evaluate their durability and operational lifespan.
- Integration into Existing Systems: Developing strategies for integrating natural ceramic membranes into existing wastewater treatment systems can ensure seamless adoption and broader impact.

## Figures and Tables

**Table 1:** A range of membrane technology indicating various operational pressures and membrane pore sizes.

Membrane Type	Pressure (Bar)	Membrane Pore Size (µm)
Reverse Osmosis (RO)	30–60	<0.001
Nanofiltration (NF)	20–40	0.001–0.005
Ultrafiltration (UF)	1–10	0.005–0.01
Microfiltration (MF)	<1	>0.01

**Table 2:** List of materials used for fabrication of oil/water separation membranes.

Membranes	Materials	Advantages	Disadvantages	Applications	Suitable Use	References
Polymeric Membranes	-Polyvinylidene fluoride (PVDF) -Polysulfone (PSf) -Polyamide (PA) -Polypropylene (PP) -Polyethersulfone (PES)	-High chemical stability -Flexible and lightweight -Relatively low cost -Easy to fabricate -High permeability	-Prone to fouling by oils and biofilms -Poor thermal stability (especially for PVDF and PSf) -Low mechanical strength compared to ceramics -Limited lifespan under harsh operating conditions	-Oil/water emulsion separation -Produced water treatment in the oil and gas industry - Wastewater treatment in industrial processes (e.g., petrochemical, food processing) -Desalination and water recycling	-Best for applications with moderate oil concentrations and mild operational conditions -Suitable for large-scale treatment systems where cost-effectiveness and easy fabrication are key	[24,25,26]
Ceramic Membranes	-Alumina (Al_2_O_3_) -Silica (SiO_2_) -Zirconia (ZrO_2_) -Titania (TiO_2_)	-High thermal and chemical stability -Superior mechanical strength -High resistance to fouling and scaling -Long lifespan -Can operate under extreme temperatures and pressures	-High manufacturing costs -Fragile, can be prone to cracking under sudden impacts or thermal cycling -High energy requirements for maintenance and cleaning -Typically, lower permeability compared to polymeric membranes	-Heavy oil/water separation in harsh environments (e.g., offshore oil rigs, chemical processing plants) -Water reuse and wastewater treatment in industrial sectors (e.g., pharmaceutical, mining) -High-performance filtration for high-temperature applications	-Best for challenging environments requiring long operational lifetimes, high resistance to fouling, and the ability to operate under high pressure or temperature conditions. -Ideal for specialised, small-scale applications where durability is paramount	[27,28,29]
Metallic Membranes	-Stainless steel -Titanium -Nickel-based alloys	-Excellent mechanical strength -High resistance to fouling and corrosion -Good stability under high temperature and pressure conditions -Can handle a wide range of pH levels	-Expensive compared to polymeric membranes -Prone to biofouling in certain conditions -Lower permeability compared to polymers	-Oil/water separation in harsh industrial environments (e.g., refineries, petrochemical plants) -Filtration of high-temperature or high-pressure fluids	Best for high-stress, extreme-temperature, and high-corrosion environments where mechanical strength and stability are essential	[30,31]
Composite Membranes	-Polymer-ceramic composite (e.g., PVDF/ceramic) -Polysulfone/ceramic -Polymeric blends with nano-scale materials (e.g., graphene, carbon nanotubes)	-Enhanced mechanical strength and stability (compared to pure polymeric membranes) -Improved resistance to fouling and scaling -Tailored properties (e.g., combining high permeability with enhanced rejection capabilities)	-Complex fabrication process -Relatively high cost -May still face challenges with biofouling	-High-performance oil/water separation applications where both high permeability and durability are required -Water treatment in industries such as oil and gas, food, and chemical	Ideal for applications needing enhanced performance but requiring lower cost than ceramic membranes, suitable for long-term, medium-to-high-pressure systems	[32,33,34]
Carbon-Based Membranes	-Activated carbon -Graphene oxide membranes (GOMs) -Carbon nanotube membranes	-Exceptional adsorption capacity for oily compounds -High selectivity and efficiency for oil removal -Low energy consumption compared to other membrane types -Graphene and carbon nanotube membranes are highly permeable	-Prone to fouling over time, especially in the presence of large organic molecules or suspended solids -High production costs for advanced carbon-based membranes (e.g., graphene) -Limited commercial availability for certain types of carbon-based membranes	-Efficient removal of oil from water in wastewater treatment -Industrial applications requiring fine filtration of emulsions or dissolved oils -Environmental cleanup (e.g., oil spill remediation)	Best for applications where high oil rejection and fine filtration are needed with a focus on minimizing fouling and energy consumption. Ideal for high-performance, selective filtration of oily water	[30,35,36]
Electrospun Membranes	-Polymers such as polyacrylonitrile (PAN), polyvinyl alcohol (PVA), and polyurethane (PU) -Nanofibre-based membranes	-High surface area and small pore sizes -Flexible and tuneable structure for specific applications -High efficiency in oil/water separation, especially for fine emulsions	-Limited mechanical strength -Potential for rapid fouling under high fouling conditions -Challenges in scaling up production	-Oil/water separation in environments with fine emulsions or low-concentration oils -Emerging applications in nanotechnology and environmental cleanup	Best for niche applications where high surface area and fine filtration are essential, such as in laboratory-scale or pilot-scale systems for specific oil/water separation tasks	[34,37,38]

**Table 3:** Summary of raw materials used for fabrication of oil/water separation membranes.

Material	Type	Advantages	Strength	Weakness	Sintering Temperature (°C)	Specific Applications	Reference
Kaolin	Ceramic	Abundant availability, good thermal and chemical stability, affordable.	Excellent heat resistance	Brittle, limited mechanical strength	1200–1400	Pretreatment membranes for oil–water separation.	[64,133,134]
Zeolite	Mineral-based	High surface area, excellent adsorption properties, ion exchange capacity.	High selectivity for contaminants	Can be sensitive to fouling in harsh conditions	900–1100	Adsorption-enhanced filtration and oil separation.	[82,84,135]
Apatite	Mineral (Phosphate-based)	Good chemical stability, adsorbs heavy metals and other pollutants.	Biocompatibility, versatile adsorption	Limited structural durability	1100–1300	Filtration in oil-contaminated industrial wastewater.	[50,135,136]
Pozzolan	Natural pozzolanic material	Eco-friendly, enhances durability, good binding properties.	High chemical resistance	Moderate strength, needs reinforcement	800–1000	Support materials for ceramic membranes.	[68,137]
Bauxite	Alumina-rich ore	High thermal and mechanical stability, resistant to abrasion.	Excellent durability and performance	Energy-intensive processing	1300–1600	Oil–water separation in harsh environments.	[23,101]
Quartz	Mineral (Silica)	High availability, excellent thermal and chemical stability, easy to process.	Superior permeability	Brittle, lower adsorption capability	1000–1200	Oil–water filtration for industrial and domestic applications.	[67,138,139]
